# EGAT: Extended Graph Attention Network for Pedestrian Trajectory Prediction

**DOI:** 10.1155/2021/9985401

**Published:** 2021-10-19

**Authors:** Wei Kong, Yun Liu, Hui Li, Chuanxu Wang

**Affiliations:** Qingdao University of Science and Technology, Qingdao, China

## Abstract

To improve foresight and make correct judgment in advance, pedestrian trajectory prediction has a wide range of application values in autonomous driving, robot interaction, and safety monitoring. However, most of the existing methods only focus on the interaction of local pedestrians according to distance, ignoring the influence of far pedestrians; the range of network input (receptive field) is small. In this paper, an extended graph attention network (EGAT) is proposed to increase receptive field, which focuses not only on local pedestrians, but also on those who are far away, to further strengthen pedestrian interaction. In the temporal domain, TSG-LSTM (TS-LSTM and TG-LSTM) and P-LSTM are proposed based on LSTM to enhance information transmission by residual connection. Compared with state-of-the-art methods, the model EGAT achieves excellent performance on both ETH and UCY public datasets and generates more reliable trajectories.

## 1. Introduction

Because of complexity and uncertainty of interaction between pedestrian and environment, it is difficult to predict human trajectory. Early methods [1, 2] have made some achievements in the study of human behavior by manual energy function, but these methods have poor generalization ability and are not suitable for constructing human-human interactions in crowded space. For methods in deep learning, such as Recurrent Neural Network (RNN) [3, 4] and Generative Adversarial Networks (GAN) [5, 6], the human interaction is modeled based on social pooling. Although the receptive field is improved, location information of pedestrians is lost. Moreover, the generator of GAN is designed with RNN, so methods of pedestrian trajectory prediction based on RNN and GAN are not only inefficient, but also costly.

Graph structure is a natural method to represent human interaction, which is more intuitive and effective than pooling methods. Graph Convolutional Network (GCN) based on graph data shows powerful modeling function, and it has become a new hotspot in the research of pedestrian interaction. In the graph, a node represents a pedestrian, and the connecting edge of two nodes represents the interaction between pedestrians. However, existing methods based on GCN cannot distinguish the importance of nodes because they distribute the weights of nodes equally. Due to the different influence of adjacent pedestrians on the target pedestrian in trajectory prediction, attention mechanism is more helpful to encode potential pedestrian interaction. On this basis, Graph Attention Network (GAT) [7] comes into being and has been widely applied. Kosaraju et al. [8] proposed Social-BiGAT, which relies on a graph to simulate human interaction, but does not make full use of graph representation. Huang et al. [9] and Mohamed et al. [10] introduced a flexible graph attention mechanism to improve social modeling, but only model the local interaction of close pedestrians.

**Figure 1:**
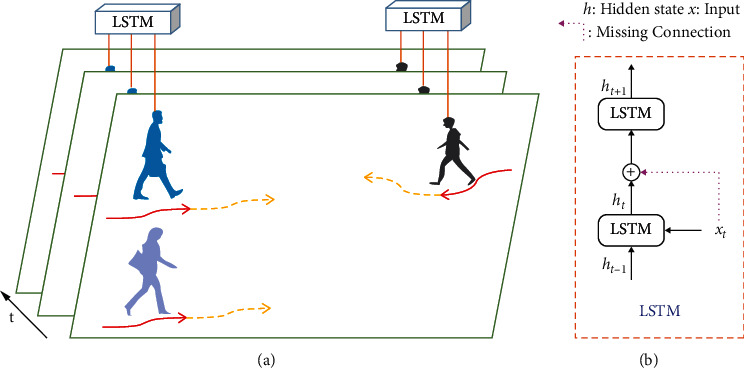
A scene of pedestrians walking. The solid red line is historical trajectory, and dotted yellow line is predicted trajectory. It is better to show in color.

At present, there are many problems in the field of pedestrian trajectory prediction. Firstly, when pedestrians are walking in a real scene, from single walking to group activity [11], social interactions are not only affected by spatial proximity. As shown in Figure 1(a), the blue pedestrian's trajectory is mainly influenced by the black people who are far away, while the purple pedestrian who is near has less influence on it.

Secondly, in the temporal domain, during modeling pedestrian's historical trajectory based on LSTM, the current state of a pedestrian only depends on the hidden state of previous moment, which ignores information transmission of the current moment and affects judgment of pedestrian's intention. See Figure 1(b) for the pink missing connection in LSTM.

Thirdly, when prediction length increases, the prediction accuracy of LSTM-based trajectory prediction models tends to decline.

EGAT is proposed in this paper to solve these problems. At first, Feature Update Mechanism (FUM) is designed in EGAT to explore global influence for pedestrians. For those far away but influential pedestrians, FUM can pay attention to them and increase the receptive field. Because the local interaction between pedestrians is extended to global interaction, the network structure is called EGAT. Next, a pedestrian's movement of next moment is mainly affected by his intention of current moment, such as going straight, turning left, or turning right Therefore, to enhance information transmission at the current time, the residual connection (i.e., the missing connection in Figure 1(b)) is added to LSTM to form TSG-LSTM (TS-LSTM and TG-LSTM). TS-LSTM and TG-LSTM models' temporal correlation for individual and interaction, respectively, not only simulates the real scene, but also reflects human dynamic movement. Then, P-LSTM predicts pedestrian trajectory based on the observed trajectory. Different from LSTM, residual connection is also added to P-LSTM. As the prediction length increases, P-LSTM alleviates prediction accuracy decreases.

## 2. Related Work

This section mainly introduces the content involved in EGAT, including human-human interactions, trajectory prediction based on RNN or attention mechanism, and application of GCN. The relevant literature of each part is compared, and the advantages of our model are put forward.

### 2.1. Human-Human Interactions

Early human interaction is defined by [1] as a social force with attraction and repulsion, which is an effective method. Due to the influence of objective environment, human-human interactions become more complex. The early models are not enough to simulate these interactions and have poor environment adaptability. On this basis, the subsequent research methods [12, 13] consider more manual rules and functions, but limit the improvement of accuracy. With the development of deep learning, complex group activities are concerned. Bagautdinov et al. [14] proposed to recognize group activity through human-human interactions. Xu et al. [15] defined pedestrian relationship based on spatial affinity. Alahi et al. [3] proposed social LSTM to aggregate interactions through social pooling. These methods only model pedestrian local interaction based on distance. In this paper, EGAT can not only predict the trajectory of a single person, but also predict potential human-human influences. It is not limited to nearby pedestrians for a target pedestrian, but focuses on all other non-local pedestrians.

### 2.2. Trajectory Prediction Based on RNN

In recent years, RNN and its variants, LSTM and GRU, have been widely used in the field of trajectory prediction. The models share parameters and show good performance. Liu et al. [16] proposed spatiotemporal RNN, which has a transformation matrix to model spatiotemporal context in each layer. Gupta et al. [5] added adversarial training based on social LSTM to improve performance. Zhang et al. [4] proposed SR-LSRM, which activates how to use the current intention of neighbors to iteratively refine the current state of crowd participants. Li et al. [17] also achieved good results by using GRU. Above research proves that RNN methods are very suitable for trajectory prediction. In this work, LSTM is improved and TSG-LSTM is proposed to encode observed trajectories of pedestrians at different time steps. Based on the observed trajectory, P-LSTM is used to predict future trajectory for pedestrians.

### 2.3. Trajectory Prediction Based on Attention Mechanism

Attention mechanism originates from imitating human vision and has significant effect on selection of relevant data [18]. The correlation coefficient between pedestrians and neighbors based on speed is determined by Su et al. [19]. Sadeghian et al. [6] combined with CNN to add bidirectional attention for pedestrians. Vemula et al. [20] used hidden state of EdgeRNN to calculate soft attention score and reflect importance of neighbors. However, these methods generally calculate the relationship between current pedestrian and adjacent pedestrians, ignoring the relationship with other long-distance pedestrians [21]. The purpose of this paper is to pay attention to all nodes in the graph, capture long-distance dependence, and extract more social features.

### 2.4. Application of GCN

GCN is very effective for data processing in non-Euclidean space. Its core idea is to map nodes or edges to vector space through deep learning methods, and then cluster and classify. GCN is widely used in action recognition [22], scene graph generation [23], video recognition [24], and other fields. Liang et al. [25] designed RNN on spatial graph to encode inductive deviation of pedestrian motion patterns. A directed social graph is dynamically constructed by Zhang et al. [26] to effectively obtain interactions of pedestrians. The Edge-Enhanced Graph Convolutional Neural Network (EGCN) proposed by Jeon et al. [27] is inherently scalable to graph nodes. In this model, frame sequences are constructed as a fully connected attention graph, in which pedestrian features involve interaction features and spatial location. The main contributions in this paper are summarized as follows:

- Extended Graph Attention Network (EGAT) is first proposed for pedestrian trajectory prediction, in which the Feature Updating Mechanism (FUM) is designed to solve the small receptive field of local attention.
- TSG-LSTM and P-LSTM are proposed based on LSTM to pay attention to the current feature information of pedestrians and improve prediction accuracy.
- On two open datasets ETH and UCY, the proposed model EGAT achieves superior performance as compared to previous methods.

## 3. Methods

**Figure 2:**
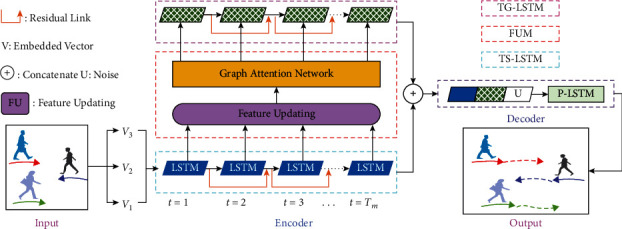
The overall framework of EGAT. It is better to show in color.

**Figure 3:**
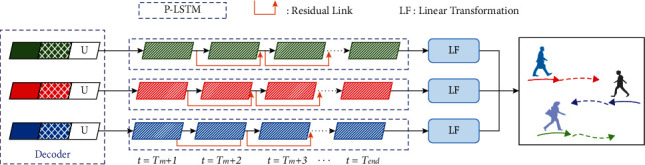
Flowchart of P-LSTM. It is better to show in color.

The structure of our proposed model is shown in Figure 2. The model consists of encoder and decoder. The encoder mainly includes FUM and TSG-LSTM (TS-LSTM and TG-LSTM). P-LSTM is a decoder. FUM, TSG-LSTM, and P-LSTM are the special designs of this paper. In Figure 2, FUM is shown in red box, TS-LSTM is shown in blue box, and TG-LSTM is shown in pink box. P-LSTM is shown in Figure 3. When encoding in the spatial and temporal domain, spatial relationship of pedestrians is encoded by FUM, while TSG-LSTM encodes the historical trajectory of pedestrians in the temporal domain. Before FUM, TS-LSTM encodes for a single pedestrian. After FUM, pedestrians already have interaction information with other pedestrians. Therefore, TG-LSTM encodes interaction relationship of pedestrians. In decoding, P-LSTM is used to predict the future trajectory of pedestrians based on encoder.

### 3.1. Problem Definition

Suppose in a scene, there are *N* pedestrians. With the movement of pedestrians, their positions are represented by two-dimensional coordinates at different times, which are used as the input of the model. At time steps 1,…, *T*_*m*_, two-dimensional coordinate of the *i*th pedestrian at each time step *t* is (*x*_*i*_^*t*^, *y*_*i*_^*t*^). The positions of *N* pedestrians during the time 1,…, *T*_*m*_ forms *N* observed trajectories; namely, *p*_*i*_^*t*^={(*x*_*i*_^*t*^, *y*_*i*_^*t*^)*|i*=1,…, *N*, *t*=1,…, *T*_*m*_}. Then, the problem is defined as follows: trajectories formed by positions of *N* pedestrians in the time interval *T*_*m*+1_,…, *T*_end_ are predicted. The definition is shown in the following equation:

(1) $$\begin{array}{c} {\hat{p}}_{i}^{t}=\left\{\left({\hat{x}}_{i}^{t},{\hat{y}}_{i}^{t}\right)|,i=1,\ldots ,N,\;t=T_{m+1},\ldots ,T_{\text{end}}\right\}. \end{array}$$

### 3.2. Encoding for a Single Pedestrian by TS-LSTM

Long Short-Term Memory (LSTM) networks have been successfully proved to be able to learn and infer attributes of a sequence, which is suitable for predicting pedestrian trajectory [3–5, 9, 28]. For observed sequences, one LSTM is denoted as TS-LSTM to encode the change of one pedestrian's movement state at different time steps.

For pedestrian *i*, firstly, the coordinate (*x*_*i*_^*t*^, *y*_*i*_^*t*^) of the pedestrian at time step *t* is embedded into a fixed length vector *v*_*i*_^*t*^ by an embedding function. The definition is shown in equation (2). Secondly, the vector is used as an input to TS-LSTM. *ω* represents an embedded function. Thirdly, TS-LSTM is used to calculate the hidden state of LSTM cell; see equation (3). *W* is a shared parameter and *h*_*i*_^*t*^ is the output. The difference between TS-LSTM and LSTM is that the residual connection is added after output. The purpose of this design is to better combine the current position feature of each pedestrian, to ensure historical information will not be lost, and achieve better information transmission.

(2) $$\begin{array}{c} v_{i}^{t}=\omega \left(x_{i}^{t},y_{i}^{t},W_{v}\right), \end{array}$$

(3) $$\begin{array}{c} h_{i}^{t}=\text{LSTM}\left(h_{i}^{t-1},v_{i}^{t},W_{h}\right)+v_{i}^{t}. \end{array}$$

### 3.3. FUM for Spatial Interaction Modeling

During pedestrian movement, the change of trajectory mainly comes from interactions of surrounding pedestrians. Therefore, it is not enough to encode a single person's motion state by TS-LSTM. To share information across pedestrians in a crowded scene, FUM is proposed in the spatial domain to treat pedestrians as nodes of a graph at each time step. FUM consists of FU and GAT. FU is the innovation to compute global interactions of nodes. GAT follows a self-attention mechanism to define the importance of neighbors. The algorithm flow of FUM is shown in Algorithm 1.

Feature Updating (FU) from equation (3): *h* only represents the features of a single pedestrian, but the interaction between pedestrians cannot be shared. To achieve global interaction and increase input range of graph attention network, FU is defined. For a target pedestrian *i*, the function of FU is to update interaction features of node *i* by implementing weighted fusion of all node features. The weight is to calculate the intimacy between node *i* and other nodes. At time *t*, relevant definitions between node *i* and node *j* are shown in the following equations:

(4) $$\begin{array}{c} M_{ij}^{t}=\frac{d\left(h_{i}^{t},h_{j}^{t}\right)}{\sum_{\forall j}d\left(h_{i}^{t},h_{j}^{t}\right)}, \end{array}$$

(5) $$\begin{array}{c} s\left(h_{j}^{t}\right)=w_{s}h_{j}^{t}, \end{array}$$

(6) $$\begin{array}{c} c_{i}^{t}=M_{ij}^{t}s\left(h_{j}^{t}\right), \end{array}$$

(7) $$\begin{array}{c} z_{i}^{t}=w_{z}c_{i}^{t}+h_{i}^{t}. \end{array}$$

**Figure 4:**
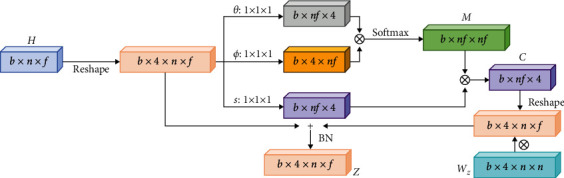
The structure of FU to calculate Z. *H* is the initial input from equation (3). *b* is the sequence length and *n* is the number of pedestrians. *f* is the embedded dimension, which is defined as 32 dimensions. 4 represents the number of attention heads. *θ*, *ϕ*, and *s* are convolution operations. *θ* and *ϕ* calculate the intimacy between nodes, that is, the weight of adjacent nodes. *s* is a display function, which is used to calculate the feature of adjacent nodes. + and ⊗ represent addition and multiplication of matrices, respectively. BN is normalization. It is better to show in color.

*d*(*h*_*i*_^*t*^, *h*_*j*_^*t*^) is a function to calculate the intimacy for any two nodes, so it increases the receptive field of the model. *s*(*h*_*j*_^*t*^) is a display function to compute the features of node *j*. The final output *z*_*i*_^*t*^ is defined by residual connection. *w*_*s*_ and *w*_*z*_ are weight parameters to learn. There are four definitions of *d*(*h*_*i*_^*t*^, *h*_*j*_^*t*^) in equation (8) and ablation experiments in Section 4.2 to verify their effectiveness. The detailed calculation of *Z* is shown in Figure 4, *Z*={*z*_*i*_^*t*^*|*∀ *i* ∈ {1,…, *N*},  *t*=1,…, *T*_*m*_}.

(8) $$\begin{array}{c} d\left(h_{i},h_{j}\right)=e^{{\left(h_{i}\right)}^{T}h_{j}},\\ d\left(h_{i},h_{j}\right)=e^{\theta {\left(h_{i}\right)}^{T}\phi \left(h_{j}\right)},\\ d\left(h_{i},h_{j}\right)=\theta {\left(h_{i}\right)}^{T}\varphi \left(h_{j}\right),\\ d\left(h_{i},h_{j}\right)=\text{ReLU}\left(w_{d}^{T}\left[\theta {\left(h_{i}\right)}^{T}\varphi \left(h_{j}\right)\right]\right). \end{array}$$

**Figure 5:**
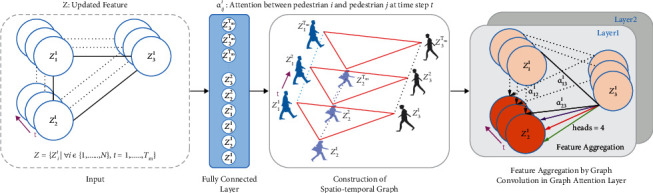
The detailed diagram of GAT on the current batch.

*Graph Attention Network*. In the spatial domain, for a graph *G* (*P*, *L*), *P* represents the set of pedestrians, *P*={*p*_*i*_^*t*^*|i*=1,…, *N*,  *t*=1,…, *T*_*m*_}. *N* is the number of pedestrians; a pedestrian is one node in *G*. *L*={*l*_*ij*_^*t*^*|*∀ *ij* ∈ {1,…, *N*},  *t*=1,…, *T*_*m*_} represents the human-human interaction at time step *t*. If there is a connection between two pedestrians, *l*_*ij*_^*t*^ equals 1, otherwise 0. The adjacency matrix *A* is constructed according to whether there are connecting edges among pedestrians, *A* ∈ *R*^*N*×*N*^. Because a fully connected graph is constructed at time *t* and all pedestrians are assumed to be connected, therefore if node *j* is a neighbor of *i*, *A*_*ij*_=1, otherwise 0. In the temporal domain, there are connecting edges for pedestrians with the same ID. Given an observed sequence, through spatial-temporal construction, the relationship of pedestrians formed a spatiotemporal graph in Figure 5. For a spatial graph *G* at time step *t*, features of pedestrians are aggregated by graph convolution. Figure 5 also illustrates the process of graph convolution and the distribution of attention.

Graph convolution network has many convolution layers. In the process of one-layer graph convolution, suppose *Z*^(*l*)^ ∈ *R*^*N*×*D*_*l*_^ represents the feature matrix of *N* pedestrians at the *l*th layer, and *D* is the feature dimension. Output of graph convolution can be written as equation (9), where $\hat{A}=A+I$, *I* is a self-connected matrix, and *σ* is an activation function. The function of trainable weight matrix *W* is to transform the dimension, *W* ∈ *R*^*D*_*l*_×*D*_*l*+1_^.

(9) $$\begin{array}{c} Z^{\left(l+1\right)}=\sigma \left(\hat{A}Z^{\left(l\right)}W\right). \end{array}$$

It can be seen from equation (9) that the adjacency matrix *A* is only used to define whether there is a connection between two nodes and cannot explain connection strength. Therefore, an attention matrix *B* needs to be defined to show the connection strength of any two nodes. During observed period, *Z*={*z*_*i*_^*t*^ ∈ *R*^*D*^*|*∀ *i* ∈ {1,…, *N*},  *t*=1,…, *T*_*m*_} is fed to a graph convolution layer. The attention coefficient of the node pair (*i*, *j*) can be computed by the following equation:

(10) $$\begin{array}{c} \alpha_{ij}^{t}=\text{softmax}\left(\exp\left(\text{Leaky ReLU}\left(a^{T}\left[W^{t}z_{i}^{t}\left\|\right)W^{t}z_{j}^{t}\right]\right)\right)\right), \end{array}$$

where *T* represents transposition, *a* ∈ *ℝ*^2*D*′^ is the weight vector of single-layer perceptron, **W**^*t*^ ∈ *ℝ*^*D*′×*D*^, || is concatenation operation, and *𝒩*_*i*_ represents neighbors of node *i* in the graph. At time step *t*, the attention matrix *B*^*t*^ ∈ *R*^*N*×*N*^ is formed by *α*_*ij*_^*t*^ (*i*, *j*=1,…, *N*), *B*={*B*^*t*^*|t*=1,…, *T*_*m*_}.

**Figure 6:**
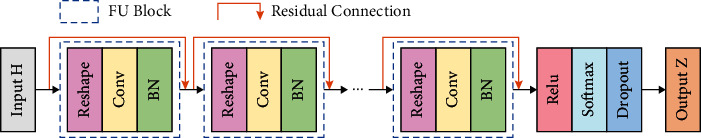
FUM is a stack of many FU blocks. Residual connection is added between blocks. It is better to show in color.

*Output of FUM*. For the observed sequence, after graph attention convolution, the final output of FUM shown in equation (11) is a softmax operation corresponding to a node *i* (*i*=1,…, *N*), ⊙ represents the multiplication of elements. *z*_*i*_^*t*^ in *Z*^(*l*+1)^ is concatenated by multi-head attention. The number of attention heads is 4. FUM can have multiple FU blocks, as shown in Figure 6, and ablation experiments in Section 4.2 to determine the number of blocks.

(11) $$\begin{array}{c} Z^{\left(l+1\right)}=\sigma \left(\rho_{i}\left(\hat{A}⊙B\right)Z^{\left(l\right)}W\right). \end{array}$$

### 3.4. TG-LSTM for Temporal Interaction Modeling

After FUM, pedestrians already have interactive information in the spatial domain. However, it is still necessary to encode the historical trajectory of pedestrian in the temporal domain. Similar to TS-LSTM, TG-LSTM is proposed. In this way, the spatial and temporal information can be fused. The definition of TG-LSTM is shown in the following equation:

(12) $$\begin{array}{c} g_{i}^{t}=LSTM\left(g_{i}^{t-1},z_{i}^{t},W_{g}\right)+z_{i}^{t}. \end{array}$$

*z*_*i*_^*t*^ is the input and comes from equation (11), *W*_*g*_ is a shared weight of TG-LSTM, and *g*_*i*_^*t*^ is the output.

Affected by the surrounding complex environment, pedestrian trajectory is uncertain. To simulate pedestrian trajectory in a real environment, in the process of training, noise *u* is randomly sampled from the standard normal distribution *N* (0,1) for each pedestrian. In complex interaction scenarios, trajectory prediction depends not only on the target pedestrian himself, but also on historical movements of surrounding pedestrians. Then, a single motion state in TS-LSTM, interactive state in TG-LSTM, and the noise *u* are concatenated to complete encoding. Therefore, at time step *t*, the observed trajectory is finally encoded as follows:

(13) $$\begin{array}{c} e_{i}^{t}=h_{i}^{t}\left\|g_{i}^{t}\right\|u. \end{array}$$

### 3.5. P-LSTM for Trajectory Prediction

For pedestrian trajectory prediction, the current state of a pedestrian can reflect his movement intention in the future. To enhance information dependence at the current moment, residual connection is also required. This can not only improve prediction performance, but also alleviate the problem that the prediction accuracy decreases when the prediction length increases. The structure of P-LSTM shows the trajectory of three pedestrians in Figure 3.

The relevant definitions are shown in equations (14) and (15), where *e*_*i*_^*T*_*m*_^ is the initial state of P-LSTM, which is derived from equation (13). *v*_*i*_^*T*_*m*_^ is from equation (2), *W*_*e*_ is an updatable weight, *δ*_*e*_ represents multilayer perception operation, and (*x*_*i*_^*T*_*m*+1_^, *y*_*i*_^*T*_*m*+1_^) is the predicted position.

(14) $$\begin{array}{c} e_{i}^{T_{m+1}}=\text{LSTM}\left(e_{i}^{T_{m}},v_{i}^{T_{m}},W_{e}\right)+v_{i}^{T_{m}}, \end{array}$$

(15) $$\begin{array}{c} \left(x_{i}^{T_{m+1}},y_{i}^{T_{m+1}}\right)=\delta_{e}\left(e_{i}^{T_{m+1}}\right). \end{array}$$

### 3.6. Definition of Loss Function

To make pedestrians respond to changes of environment and improve the accuracy of trajectory prediction, the diverse loss method proposed by Gupta et al. [5] simulates the polymorphism of pedestrian movement. The definition of loss is shown in equation (16). During training, different Gaussian noise *u* is sampled to produce *k* results in one prediction. L2 distance is calculated *k* times, and the minimum value is taken as the loss. *Y*_*i*_ is the actual trajectory, ${\hat{Y}}_{i}$ is the predicted trajectory, and *k* is a super parameter. In this paper, *k* = 20.

(16) $$\begin{array}{c} L=\min_{k}{\left\|Y_{i}-{\hat{Y}}_{i}^{k}\right\|}_{2}. \end{array}$$

## 4. Experiments and Results Analysis

In this section, Section 4.1 first introduces the experimental settings. Next, ablation experiments for FUM and residual connection are displayed in Section 4.2. Then, our model EGAT is compared with other models in Section 4.3. Finally, experimental results of our proposed model are analyzed in Section 4.4.

### 4.1. Experiment Settings

The experiment settings include datasets, evaluation metrics, and implementation details.

#### 4.1.1. Datasets

The model is experimented on two pedestrian trajectory datasets: ETH [12] and UCY [29]. ETH includes two scenes: ETH and HOTEL. UCY consists of three scenes: ZARA1, ZARA2, and UNIV. The original dataset of each scene is a video shot from an aerial view, which involves many complex situations, such as pedestrians walking, pedestrians staying talking, and complex environment. These datasets have 2206 human motion trajectories. All the data has been converted to world coordinates and the trajectory is sampled every 0.4 seconds. When training on five scene datasets, following previous studies [3, 5, 9], the leave-one-out method is adopted. The model is trained on four scenes, and the remaining one is tested. The observed trajectory is 3.2 seconds (8 time steps), and the predicted trajectory is 4.8 seconds (12 time steps).

#### 4.1.2. Evaluation Metrics

There are two metrics to evaluate the model's performance. They are the average displacement error (ADE) and the final displacement error (FDE). Definitions are shown in equations (17) and (18). Specifically, ADE evaluates the average prediction performance, while FDE only considers the final prediction accuracy. The smaller the value of the two metrics, the better the prediction results. The two metrics are defined as follows:

(17) $$\begin{array}{c} \text{ADE}=\frac{\sum_{i\in N}\sum_{t\in T_{end}}{\left\|{\hat{p}}_{i}^{t}-p_{i}^{t}\right\|}_{2}}{N\times T_{\text{end}}}, \end{array}$$

(18) $$\begin{array}{c} \text{FDE}=\frac{\sum_{i\in N}{\left\|{\hat{p}}_{i}^{t}-p_{i}^{t}\right\|}_{2}}{N},\;t=T_{\text{end}}. \end{array}$$

#### 4.1.3. Implementation Details

The proposed network EGAT is implemented in PyTorch 1.2 framework using *Python* language, and trained with two NVIDIA GeForce GTX-1080 GPUs. The setting of learning rate in different datasets is shown in Table 1. Adam optimizer is used and batch size is 64. TSG-LSTM and P-LSTM have only one layer. The size of hidden state and output of TSG-LSTM is 32 dimensions. Embedded vector *v*_*i*_^*t*^ is 32 dimensions. FUM has two layers, and its input is normalized. The size of noise *u* is set to 16 dimensions.

#### 4.1.4. Training Visualization

**Figure 7:**
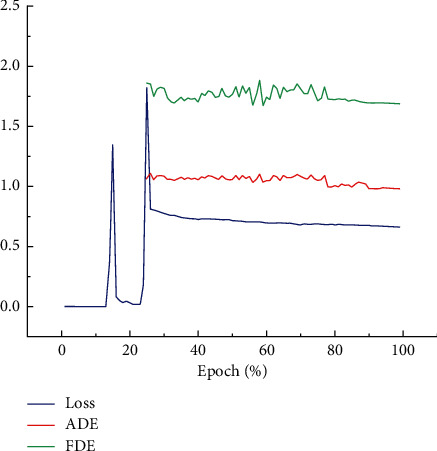
When training to 25%, Loss, ADE, and FDE are gradually fitted. It is better to show in color.

The trends of Loss, ADE, and FDE during training are shown in Figure 7. The change of Loss shows that the training process is divided into three stages: 15% of epochs are used to encode for a single pedestrian by TS-LSTM, 15% to 25% of epochs are trained for FUM and TG-LSTM, and the remaining epochs are decoded based on the previous encoding to predict trajectory. When the epoch is less than 25% in the process of training, the model is encoding and has not predicted the trajectory. In this case, the displacement error between the predicted trajectory and the ground truth cannot be calculated. The error is calculated by ADE and FDE. Therefore, when the epoch is less than 25%, ADE and FDE have no curves in Figure 7.

### 4.2. Ablation Study

In this section, the ablation experiments of FUM and residual connection are carried out. For FUM, the intimacy function and FU blocks are studied. For residual connection, the experimental performance of TSG-LSTM and P-LSTM is verified, and the influence of residual connection on the model is compared on all datasets.

#### 4.2.1. Ablation Study of FUM

To evaluate the effectiveness of FUM, the ablation experiments are as follows.

1. *Baseline*. STGAT-20V-20 is directly applied to predict pedestrian trajectory without FUM and LSTM residual connection, and the prediction length is 8.
2. *Intimacy Function*.  Table 2 compares effects of *d*(*h*_*i*_, *h*_*j*_) with different structures on the model performance. Because embed-Gaussian uses softmax function, it can directly give the fraction between [0,1], and its experimental effect is the best.
3. *FU Blocks*. In each layer of FUM, multiple FU blocks can be set to calculate extended features. The ablation experiments in Table 2 based on embed-Gaussian function show that the model performs best when the number of blocks is 4. As blocks increases, the performance decreases. This is because the node information can be transmitted back and forth in a long distance. After more blocks, the feature information becomes smooth.

#### 4.2.2. Temporal Residual

In the output of TSG-LSTM and P-LSTM, residual connection is designed separately to enhance the transmission and combination of feature information. Six methods are compared in Table 3.

1. *Baseline*. STGAT-20V-20.
2. *FUM (4,embed-Gaussian)*. Add FUM to STGAT-20v-20. The number of FU blocks is 4, and the intimacy function is embed-Gaussian. The following abbreviation is FUM.
3. *FUM + TS-LSTM*. TS-LSTM adds residual connection to LSTM to encode the trajectory of a single pedestrian in the temporal domain. However, TS-LSTM ignores pedestrian interaction information.
4. *FUM + TG-LSTM*. TG-LSTM adds residual connection to LSTM to encode the historical trajectory of a pedestrian loaded with interactive features. Due to the complex and changeable nature of pedestrian movement, interactions appear particularly important in trajectory prediction. As shown in Table 3, experimental results of TG-LSTM are better than TS-LSTM, but the personal information is lost.
5. *FUM + TSG-LSTM*. TSG-LSTM is a combination of TS-LSTM and TG-LSTM. It contains not only personal information but also interactive information, so the experimental performance is further improved. The ablation results in Table 3 prove the significance of TSG-LSTM.
6. *FUM + TSG-LSTM + P-LSTM (EGAT)*. After adding FUM and residual connection, as can be seen from the last row of Table 3, our model EGAT applies P-LSTM to enhance the current information transmission of pedestrians in prediction process, so the experimental performance is the best.

#### 4.2.3. Residual Connection for All Datasets

For all datasets, Table 4 compares differences between design (EGAT) and no-design residual connections (UN-EGAT). Experimental results show that the average value of ADE and FDE can be reduced by 20% and 17% by adding residual connection. The lower the value, the better the network performance.

### 4.3. Comparison with the State-of-the-Art

The comparison between EGAT and other models is based on five scenarios of ETH and UCY, using evaluation metrics ADE and FDE with prediction length of 12. The experimental results show that the performance of the proposed EGAT model is better than most of the methods.

#### 4.3.1. Evaluation Metrics Analysis

The proposed model is compared with the state-of-the-art models in Table 5. STGAT-20V-20 is considered as the baseline model. EGAT is superior to STGAT-20V-20 in all datasets. The values of ADE and FDE in ETH and HOTEL, ADE in ZARA2, and AVG ADE are the best among the models listed in Table 5. The other values are close to the optimal values. There are two reasons why the optimal value is not reached. In UNIV, pedestrians are dense, and the environment is complex. The interaction between pedestrians is affected by many factors, such as motion speed, motion direction, motion state, and so on. These factors affect the prediction accuracy of the model. In ZARA1, the trajectory of pedestrians is often affected by the surrounding pedestrians and obstacles, which may change or limit human activities, resulting in the model being unable to capture more social interactions.

#### 4.3.2. Inference Time and Parameters

The results of all models are run on two NVIDIA GeForce GTX-1080 GPUs. As can be seen from Table 6, EGAT is superior to some models. When calculating inference time (in seconds), EGAT uses residual connection to concatenate individual state and interactive state, which makes the inference time increase. As for parameters, EGAT's parameters are slightly higher than STGAT, because the intimacy with all nodes on a graph needs to be calculated.

### 4.4. Analysis of Experimental Results

In this section, the visualization results of attention and prediction trajectory are analyzed, the existing problems are described, and the future research direction of this paper is prospected.

#### 4.4.1. Attention Visualization

**Figure 8:**
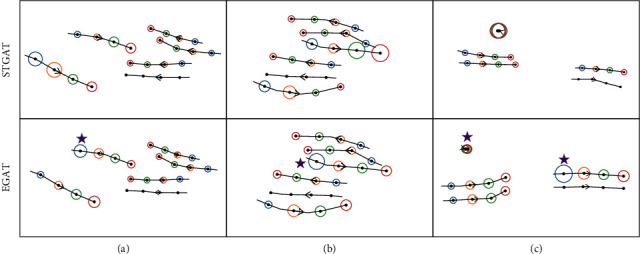
Comparison of attention in EGAT and STGAT. The line without circle is a target pedestrian, black solid points on the line represent different time steps, and the arrow indicates the direction of pedestrian movement. The circled lines are other pedestrians associated with the target pedestrian. The larger the circle, the more attention given to the pedestrian in the current time step, and the more influence on the target pedestrian's trajectory. It is better to show in color.

It is found that the difference of attention allocation between EGAT and STGAT is mainly reflected in the last four time steps. Therefore, Figure 8 compares the changes of attention in four time steps. The purple star annotates the difference between EGAT and STGAT. Through comparison, it can be found that EGAT can more successfully reflect the importance of pedestrians, which is closer to a social reality scene. In (a) and (b), the pedestrian marked by a purple star of EGAT has the greatest impact on the target pedestrian trajectory, which is more accurate than STGAT. In (c), STGAT pays more attention to a stationary pedestrian, which is contrary to reality. However, EGAT correctly judges the stationary pedestrian (left purple star), allocates small attention to it, and focuses on the movement of adjacent pedestrians (right purple star). Visualization shows that the EGAT can expand receptive field, get global feature information, and enhance information transmission.

#### 4.4.2. Predicted Trajectory

**Figure 9:**
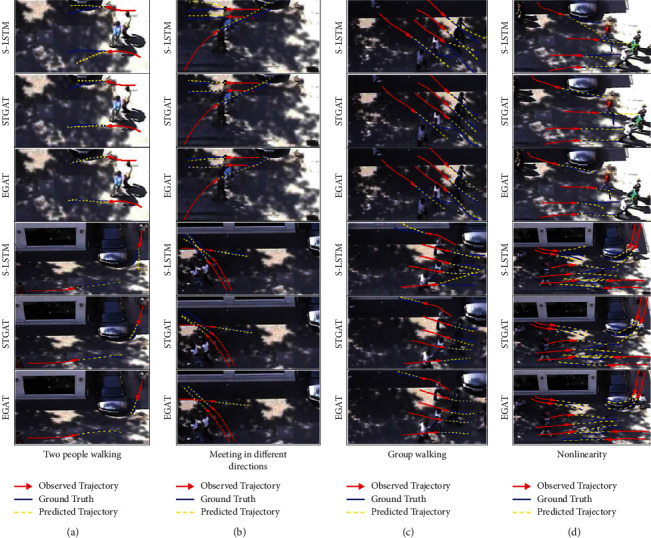
Visualization of predicted trajectory. Red solid line is the observed trajectory, blue solid line is the ground truth, and yellow dotted line is the predicted trajectory. The higher the coincidence between yellow dotted line and blue solid line, the better the prediction performance of the model. It is better to show in color.

The visual results of trajectory prediction for EGAT, STGAT, and S-LSTM are shown in Figure 9. Four scenarios are compared. It can be seen that the prediction performance of EGAT is the best among the three models. Group (a) compares the movement of two pedestrians. According to the coincidence of the ground truth and the predicted trajectory, EGAT achieves better prediction whether the two pedestrians are parallel or cross. In group (b), both EGAT and STGAT can produce reasonable trajectories to avoid collision. If the trajectory is carefully observed, EGAT's predicted trajectory is closer to the real trajectory. However, S-LSTM performed poorly. Group (c) introduces the scene of group walking, including parallel walking and meeting. Although the scene is complex, EGAT gives more accurate prediction. Group (d) focuses on the scene of nonlinear walking. In the first three pictures, from top to bottom, the second trajectory turns. EGAT successfully captures the intention of pedestrian and accurately realizes turning. STGAT and S-LSTM only realize local interaction between pedestrians, and the predicted trajectory is still straight, which makes the results different from the real trajectory. Similarly, in the following three pictures, two pedestrians next to the car can turn smoothly in EGAT, while STGAT has a certain realization, but it is not as accurate as EGAT. The trajectory generated by S-LSTM is not satisfactory. In a word, the proposed model EGAT can not only predict linear motion successfully, but also capture nonlinear motion reasonably, and its performance is better.

#### 4.4.3. Problems and Research Direction

**Figure 10:**
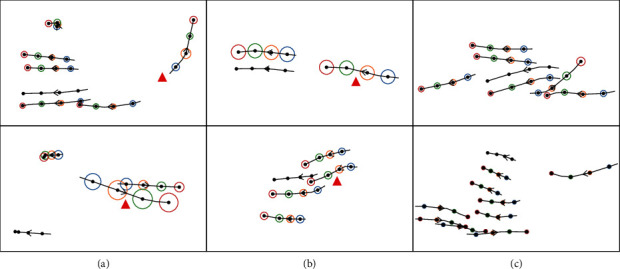
Inappropriate distribution of attention. It is better to show in color.

First, the shallow structure of GCN: experimental results show that if network layers of GCN are greater than two, the performance will decline [31]. The reason is that if layers stack too deep, features of each node in the graph will be excessively smooth. Therefore, the number of graph convolution layers is usually only two to three, the network structure cannot be deepened vertically. Second, the extension of attention is not sufficient. Due to single-feature information in datasets, although the proposed model improves receptive field, the model still focuses attention on spatial distance, and fusion of information such as walking direction and speed of pedestrians is not enough. In Figures 10(a) and 10(b), the target pedestrian generally pays more attention to pedestrians in front, while pedestrians marked by a red triangle get more attention, although they are all located behind the target pedestrian. This is mainly because the model does not combine direction information. Moreover, when there is a great number of pedestrians at the same time, the calculation of node intimacy reduces the difference between pedestrians, and it is easy to have a uniform distribution of attention, as shown in Figure 10(c). Therefore, the future research focuses on the deep exploration of graph convolution, fusion application of information, and improvement of the model generalization ability.

## 5. Conclusion

A novel EGAT framework is proposed in this paper, which can predict pedestrian trajectory in different scenes. EGAT not only improves the receptive field of the model, but also improves the prediction performance when the prediction length increases. During encoding, Graph Attention Network is extended to model human-human interactions in the spatial domain, and the historical trajectory of pedestrians is encoded by TSG-LSTM in the temporal domain. When decoding, P-LSTM predicts the pedestrian trajectory based on observed trajectories. EGAT is superior to STGAT on two public datasets. The experimental results show that EGAT can allocate reasonable weights to pedestrians according to their motion states, and the model can get more accurate trajectories.

## Figures and Tables

**Algorithm 1:**
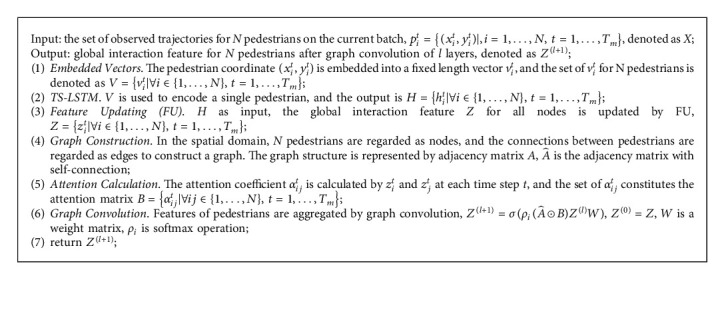
Algorithm 1 Framework of FUM to model spatial interaction.

**Table 1:** Setting of learning rate.

Dataset	Learning rate	
	Initialization	Epoch proportion ≥25%
ETH	1*e* − 6	5*e* − 3
HOTEL	1*e* − 6	9.5*e* − 3
UNIV	1*e* − 5	5*e* − 3
ZARA1	1*e* − 5	5*e* − 3
ZARA2	1*e* − 5	1*e* − 2

**Table 2:** Ablation study of FUM.

Metrics	Baseline	Intimacy function				FUM blocks (embed-Gaussian)				
		Gaussian	Embed-Gaussian	Dot-product	Concatenation	2	4	6	10	16
ADE↓	0.20	0.22	0.20	0.21	0.22	0.21	0.19	0.20	0.21	0.22
FDE↓	0.40	0.45	0.40	0.44	0.44	0.41	0.39	0.39	0.41	0.41

**Table 3:** Ablation study of temporal residual.

Methods	ADE↓	FDE↓
Baseline	0.2035	0.4047
FUM (4,embed-Gaussian)	0.1942	0.3933
FUM + TS-LSTM	0.1920	0.3914
FUM + TG-LSTM	0.1896	0.3842
FUM + TSG-LSTM	0.1830	0.3751
FUM + TSG-LSTM + P-LSTM (EGAT)	0.1805	0.3713

**Table 4:** Ablation study of residual connection for all datasets.

Dataset	ETH		HOTEL		UNIV		ZARA1		ZARA2		AVG	
Metrics	ADE↓	FDE↓	ADE↓	FDE↓	ADE↓	FDE↓	ADE↓	FDE↓	ADE↓	FDE↓	ADE↓	FDE↓
UN-EGAT	0.64	1.19	0.40	0.75	0.33	0.67	0.22	0.44	0.20	0.41	0.36	0.69
EGAT	0.50	0.95	0.26	0.49	0.31	0.65	0.20	0.41	0.18	0.37	0.29	0.57

**Table 5:** Comparison with the state-of-the-art. Top-1, Top-2, and Top-3 results are shown in red, green, and blue.

Dataset	ETH		HOTEL		UNIV		ZARA1		ZARA2		AVG	
Metrics	ADE	FDE	ADE	FDE	ADE	FDE	ADE	FDE	ADE	FDE	ADE	FDE
Linear^*∗*^ [3]	1.33	2.94	0.39	0.72	0.82	1.59	0.62	1.21	0.77	1.48	0.79	1.59
SR-LSTM-2^*∗*^ [4]	0.63	1.25	0.37	0.74	0.51	1.10	0.41	0.90	0.32	0.70	0.45	0.94
S-LSTM [3]	1.09	2.35	0.79	1.76	0.67	1.40	0.47	1.00	0.56	1.17	0.72	1.54
S-GAN-P [5]	0.87	1.62	0.67	1.37	0.76	1.52	0.35	0.68	0.42	0.84	0.61	1.21
SoPhie [6]	0.70	1.43	0.76	1.67	0.54	1.24	0.30	0.63	0.38	0.78	0.54	1.15
CGNS [17]	0.62	1.40	0.70	0.93	0.48	1.22	0.32	0.59	0.35	0.71	0.49	0.97
PIF [30]	0.73	1.65	0.30	0.59	0.60	1.27	0.38	0.81	0.31	0.68	0.46	1.00
STSGN [26]	0.75	1.63	0.63	1.01	0.48	1.08	0.30	0.65	0.26	0.57	0.48	0.99
GAT [8]	0.68	1.29	0.68	1.40	0.57	1.29	0.29	0.60	0.37	0.75	0.52	1.07
Social-BiGAT [8]	0.69	1.29	0.49	1.01	0.55	1.32	0.30	0.62	0.36	0.75	0.48	1.00
Social-STGCNN [10]	0.64	1.11	0.49	0.85	0.44	0.79	0.34	0.53	0.30	0.48	0.44	0.75
STGAT-20v-20 [9]	0.65	1.12	0.35	0.66	0.52	1.10	0.34	0.69	0.29	0.60	0.43	0.83
EGAT	0.57	1.03	0.30	0.58	0.50	1.09	0.33	0.65	0.26	0.57	0.39	0.78

**Table 6:** Inference time and parameters in different models.

Methods	Inference time (s)	Parameters (K)
S-LSTM [3]	1.4736	264
SR-LSTM [4]	0.1973	64.9
S-GAN-P [5]	0.1210	46.3
PIF [30]	0.1431	360
Social-STGCNN [10]	0.0025	7.6
STGAT [9]	0.0310	44.63
EGAT	0.0492	44.7

## Data Availability

The data used to support the findings of this study are available from the corresponding author upon request.
